# ICAM-1 Targeted Nanogels Loaded with Dexamethasone Alleviate Pulmonary Inflammation

**DOI:** 10.1371/journal.pone.0102329

**Published:** 2014-07-14

**Authors:** M. Carme Coll Ferrer, Vladimir V. Shuvaev, Blaine J. Zern, Russell J. Composto, Vladimir R. Muzykantov, David M. Eckmann

**Affiliations:** 1 Department of Anesthesiology and Critical Care, University of Pennsylvania, Philadelphia, Pennsylvania, United States of America; 2 Department of Pharmacology and Center for Targeted Therapeutics and Translational Nanomedicine, University of Pennsylvania, Philadelphia, Pennsylvania, United States of America; 3 Materials Science and Engineering, University of Pennsylvania, Philadelphia, Pennsylvania, United States of America; Helmholtz Zentrum München, Germany

## Abstract

Lysozyme dextran nanogels (NG) have great potential *in vitro* as a drug delivery platform, combining simple chemistry with rapid uptake and cargo release in target cells with “stealth” properties and low toxicity. In this work, we study for the first time the potential of targeted NG as a drug delivery platform *in vivo* to alleviate acute pulmonary inflammation in animal model of LPS-induced lung injury. NG are targeted to the endothelium via conjugation with an antibody (Ab) directed to Intercellular Adhesion Molecule-1(ICAM-NG), whereas IgG conjugated NG (IgG-NG) are used for control formulations. The amount of Ab conjugated to the NG and distribution in the body after intravenous (IV) injection have been quantitatively analyzed using a tracer isotope-labeled [^125^I]IgG. As a proof of concept, Ab-NG are loaded with dexamethasone, an anti-inflammatory therapeutic, and the drug uptake and release kinetics are measured by HPLC. *In vivo* studies in mice showed that: i) ICAM-NG accumulates in mouse lungs (∼120% ID/g vs ∼15% ID/g of IgG-NG); and, ii) DEX encapsulated in ICAM-NG, but not in IgG-NG practically blocks LPS-induced overexpression of pro-inflammatory cell adhesion molecules including ICAM-1 in the pulmonary inflammation.

## Introduction

The endothelial monolayer lining the vasculature represents a multifunctional regulatory interface between blood and tissues [1]–[5]. Endothelial abnormalities are implicated in the pathogenesis of cardiovascular, neurological, pulmonary, metabolic, and other conditions [6]–[8]. In these conditions, endothelial cells represent an important participant, victim and therapeutic target [9]–[12]. In particular, the pulmonary endothelium is an important target for treatment of acute inflammation, such as acute lung injury/acute respiratory distress syndrome [1].

Acute lung injury causes disruption of the lung endothelial and epithelial barriers. As a consequence, the lungs mechanics change (i.e., lungs become stiffer) and the number of pores media available for gas exchange are compromised. Most current treatments involve ventilatory strategies, which further traumatize the lung. Other pharmacological treatments attempted in clinical trials have yet not been effective in reducing mortality [13]. In the US, the incidence of acute lung injury is estimated at 200,000 cases with a mortality rate of 40% and is mainly associated with intensive care unit disorders such as sepsis, pneumonia and trauma [14].

Most drugs and drug carriers have no natural affinity to endothelium [15], [16]; hence only a minor fraction of the dose acts in this target, despite its accessibility to the bloodstream. As a result, systemic drug delivery and effective pharmacotherapies intended to treat abnormalities of pulmonary endothelium are not sufficient to cope with acute grave disorders like acute lung injury/acute respiratory distress syndrome. In order to achieve this goal, we conjugate drugs and drug carriers with antibodies and other affinity ligands that bind to endothelial cells [17]–[19]. Pulmonary vasculature represents ∼25% of the total endothelial surface and receives essentially the entirety of the right-sided cardiac output; hence these compounds targeted to the endothelium accumulate in the lungs [20]–[22].

Surface receptors of endothelial cells include intracellular adhesion molecules (ICAM-1), a transmembrane glycoprotein. Its antibody, Anti-ICAM-1, is known to accumulate in the lungs after intravenous (IV) injection and has been used for drug targeting to the endothelium [23], [24].

Dexametasone (DEX) is a potent long lasting synthetic glucocorticoid known to inhibit the inflammatory cascade. DEX mainly acts by suppressing expression of proinflammatory cytokines (IL-1, IL-6, IL-8 and TNF-α) and cell adhesion molecules (endothelial leucocyte adhesion molecule-1 and ICAM-1) involved in the migration of leucocytes into the extravascular space [25]. Although DEX is utilized frequently in hospital and out-patients to relieve inflammation in different parts of the body including the lungs, DEX can cause systematic side effects. Consequently, efforts have focused on delivery DEX via drug delivery system such as immunoconjugates [26], polymeric nanocarriers [27] and liposomes [28]. Alternatively, we proposed to deliver DEX locally to the inflammation site via a nanogel system.

Nanogels are nanosized networks that can absorb large amounts of water while preserving their structure via physical or chemical crosslinks [29], [30]. In the swollen state, nanogels behave as soft gels known to minimize non-specific interactions with *fluid-like transport properties*. In contrast to traditional nanoparticles (i.e., stiffer gels/nanocarriers), nanogels can deform to pass physiological filters resulting in greater delivery efficiency than can be reached using stiffer nanoparticles. Additionally, nanogels overcome limitations of other delivery systems such as liposomes. These include limited drug loading capacity of immunoconjugates, greater stability, easiness for sterilization and lower clearance due to non-specific uptake.

In previous work, we developed biocompatible nanogels composed of a rhodamine-labeled dextran shell and a lysozyme core (NG) and examined their viability for drug delivery with a fluorescent molecular probe as a mock drug in two *in vitro* models. Human umbilical vein endothelial cells (HUVEC) were used as a cell culture model to verify NG uptake, drug release and assess cytotoxicity whereas differentiated macrophages (THP-1 cells stimulated with PMA) were used as a model of the mononuclear phagocyte system. These NG showed great potential based on their lack of cytotoxicity and rapid release of the drug in HUVEC before reaching the lysosomes, as compared to their slow uptake by macrophages [31]. In other work, we also highlighted the potential of similar NG for antimicrobial therapy applications when loaded with Ag NPs by *in situ* reaction in the NG solution [32], [33].

In this study, we aim to enhance delivery of DEX to the lungs while reducing the toxicity of free DEX to non-target organs. This is accomplished by grafting these biocompatible NG with anti-ICAM (ICAM-NG) directed to the pulmonary endothelium. Naïve mice are used to verify *in vivo* targeting of ICAM-NG. Furthermore, ICAM-NG are loaded with DEX (ICAM-NG-DEX), and their potential to alleviate pulmonary inflammation is studied in a mouse model of inducible inflammatory state. As control formulations, IgG-conjugated NG (IgG-NG) are used.

## Materials and Methods

### Materials

Rhodamine B isothiocyanate–dextran from *Leuconostoc* ssp. (64–76 kDa molecular weight), lysozyme from chicken egg white, lipopolysaccharides (LPS, from *E. coli* O55:B5) and dexamethasone (DEX) were obtained from Sigma-Aldrich (St. Louis, MI). Anti-ICAM monoclonal antibody (mAb) used was mAb YN1/1.7.4, a rat mAb directed against murine Intercellular Adhesion Molecule-1 (ICAM) [34]. Rat IgG was purchased from Equitech Bio, Inc. (Kerrville, TX). Millipore water (18.2 MΩ.cm) was used.

### Methods

#### DLS analysis

The particle size and size distribution of the hydrated NG was determined by dynamic light scattering using a ZS90 Malvern Zetasize Nano series instrument (Malvern, Westborough, MA) equipped with a 22 mW He-Ne laser operating at a wavelength of 633 nm.

#### Cryo-TEM analysis

Nanogel morphology was imaged by cryo-transmission electronic microscopy on a FEI tecnai-12 operated at 120 keV. The images were recorded on a Gatan ultrascan 1000 CCD camera (2048×2048 pixels, with each pixel dimension at 14 µm).

#### γ-Counter analysis

Radioactivity was determined using 2470 WIZARD^2^ Automatic Gamma Counter (PerkinElmer Inc., Waltham, MA). The amount of dexamethasone released was analyzed using HPLC (Beckman-Coulter, Inc., Brea, CA). Analysis was performed at 246 nm using a Beckman-Coulter 250 mm reversed-phase ultrasphere ODS column with a mobile phase of 40% 2 mM acetate buffer (pH 4.8) and 60% acetonitrile flowing at 1 mL min^−1^ at room temperature.

### Synthesis of NG, conjugation of antibodies (Ab) and loading of dexamethasone

The synthesis of NG was performed as previously reported [31], [35]. Briefly, Rhodamine B isothiocyanate–dextran and lysozyme were dissolved at 1∶1 molar ratio in water, the pH was adjusted to between 7 and 8 using 0.1 N sodium hydroxide and the solution was lyophilized. The lyophilized powder was allowed to react at 60°C under 79% relative humidity in a desiccator containing saturated KBr solution for 18 to 24 h. The reacted powder was dissolved in water (5 mg/mL, based on dextran and lysozyme together), the pH was adjusted to 10.7 using 0.1 N sodium hydroxide, and the solution was further reacted at 80°C for 30 min. The NG were stored at 4°C.

**Figure 1:**
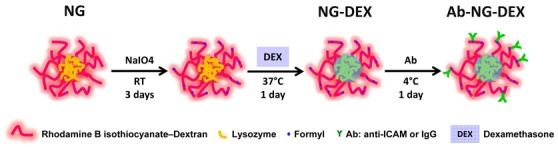
Schematic illustrating the conjugation of Ab to NG and loading of DEX.

A schematic showing the conjugation of antibodies (Ab) to the NG (Ab-NG) and loading of DEX (Ab-NG-DEX) is illustrated in Figure 1. Prior to Ab conjugation, the NG were activated using sodium periodate (NaIO_4_) under mild conditions (i.e., room temperature, 3 days) at 1∶0.01 molar ratio of dextran∶NaIO_4_ to generate formyl groups [36] and when required, the NG were loaded with DEX. To do so, NG (400 µL) were concentrated to 35 µL by ultracentrifugation (Amicon, Millipore, Billerica, MA), dispersed with 100 µL NaIO_4_ 0.01 M, 100 µL NaCl 25 wt% and 265 µL deionized water, and allowed to react over 3 days on a shaker at room temperature in the dark. Following reaction, the NG were spun down and dispersed to 500 µL with deionized water twice sequentially. To load them with DEX (NG-DEX), NG (0.22 wt. %) were incubated on a shaker with DEX at a final concentration of 645 µg/mL for 1 day at 37°C. For conjugation, 100 µg of Ab (either IgG (IgG-NG) or anti-ICAM (ICAM-NG), in PBS containing ≤0.09% sodium azide) was added to 300 µL of NG (or NG-DEX) suspension in DI water and placed on a shaker overnight at 4°C. Following Ab conjugation, Ab-NG (or Ab-NG-DEX) mixture was centrifuged at 16,000×g for 15 min to remove unbound Ab (and free DEX). The Ab-NG (or Ab-NG-DEX) pellet was then dispersed via sonication into 1 wt% BSA/PBS.

### Anti-ICAM coating efficiency of ICAM-NG

The ICAM-NG were formulated with a trace amount of conjugated [^125^I]IgG (∼2 µCi) in 200 µL 1 wt% BSA/PBS following same procedure described above. The anti-ICAM coating efficiency was determined by measuring [^125^I]IgG conjugated onto NG relative to the IgG mass added. Briefly, ICAM-NG were centrifuged at 16,000×g for 15 min to remove unbound [^125^I]IgG. The ICAM-NG was then dispersed via sonication into PBS. The coating efficiency was then calculated as [^125^I]IgG free/[^125^I]IgG bound [37]. The number of IgG molecules per NG was calculated based on NG surface area, (A = 4πr^2^, assuming NP diameter is 105 nm) and treating mAb as a block (footprint equates to ∼120 nm^2^). Ultimately, this corresponded to ∼250 mAbs/NP.

### In vitro release kinetics of DEX from IgG-NG-DEX

Fresh purified IgG-NG-DEX pellet dispersed via sonication into 1 wt% BSA/PBS was placed on a shaker at 37°C for DEX release kinetics study. At each time point studied, the IgG-NG-DEX were centrifuged at 14,000×g for 15 min to remove free DEX, redispersed via sonication into 1 wt% BSA/PBS and returned to the shaker at 37°C. The concentration of free DEX in the natants, as well as the drug encapsulation efficiency, was measured by HPLC. To account for the free DEX that may have released out of IgG-NG-DEX while preparing them for injection into mice (i.e., under static conditions at room temperature), fresh purified IgG-NG-DEX pellet were allowed to sit at room temperature for 30 min before centrifugation to obtain a measure of free DEX drug loss. This amount was proportionately subtracted from the 15 min and 30 min time points. The corrected drug encapsulation efficiency of IgG-NG-DEX was 17%. The experimental data were fitted to an extended Langmuir model (LangmuirEXT1, Simplex) with OriginPro software (Northampton, MA).

### In vivo studies

All animal studies were carried out in strict accordance with the recommendations in the Guide for the Care and Use of Laboratory Animals of the National Institutes of Health. The protocol was approved by the Committee on the Ethics of Animal Experiments of the University of Pennsylvania (Permit Number: 803375). All surgery was performed under ketamine/xylazine anesthesia, and all efforts were made to minimize suffering.

### Biodistribution studies of Ab-NG in mice

This study was performed on 6 to 8 week-old female C57BL/6 mice (18 to 22 g; *n* = 3 to 5 per group). Mice were anesthetized and injected IV via the jugular vein with ICAM-NG or IgG-NG. Mice were injected with approximately 2.2 mg of Ab-NG formulated with a trace amount of conjugated [^125^I]IgG (∼2 µCi) in 200 µL 1 wt% BSA/PBS. At 30 min post-injection of Ab-NG, blood was collected from the retro-orbital sinus, the animals were euthanized and organs (heart, kidneys, liver, spleen, lungs, and brain) were extracted, weighed and blotted. Tissue radioactivity was measured in a γ-counter, and NG targeting parameters defined by percent of injected dose per gram of tissue (% ID/g). The total activity in blood was calculated assuming a total blood volume representing 7% of the mouse body weight.

### Endotoxemia model in mice

This study was performed on 6 to 8 week-old male C57BL/6 mice (20 to 25 g, *n* = 5 to 7 per group). Mice were anesthetized and either 2.2 mg of ICAM-NG-DEX or control (IgG-NG-DEX, ICAM-NG or 1 wt% BSA/PBS) in approximately 200 µL was injected 15 min prior to LPS administration (200 µg/kg) via the tail vein [38]. After 24 h elapsed from initiation of the LPS challenge, the mice were euthanized and the lungs were perfused and harvested. Lungs were homogenated and VCAM-1 and ICAM-1 expression levels were assayed by Western blot.

### Western blot analysis

Lung homogenates were subjected to 4–15% gradient gel. Gels were transferred to PVDF membranes (Millipore) and the membranes were blocked with 3% nonfat dry milk in TBS-T (100 mM Tris (pH 7.5), 150 mM NaCl, 0.1% Tween 20) for 1 h. Proteins were detected using goat polyclonal anti-VCAM (R&D Systems, Minneapolis, MN) and anti-ICAM (Santa Cruz Biotechnology, Inc., Santa Cruz, CA), and anti-actin-HRP (Abcam, Cambridge, MA).

### Statistical analysis

Data are presented as mean ± SD, unless otherwise noted. Statistical significance between two groups was assessed via t-test, while multiple groups were compared via ANOVA followed by Student's t-test (OriginLab, Northampton, MA). Values of *p*<0.05 were considered statistically significant.

## Results

### Synthesis and characterization of Ab conjugated NG

**Figure 2:**
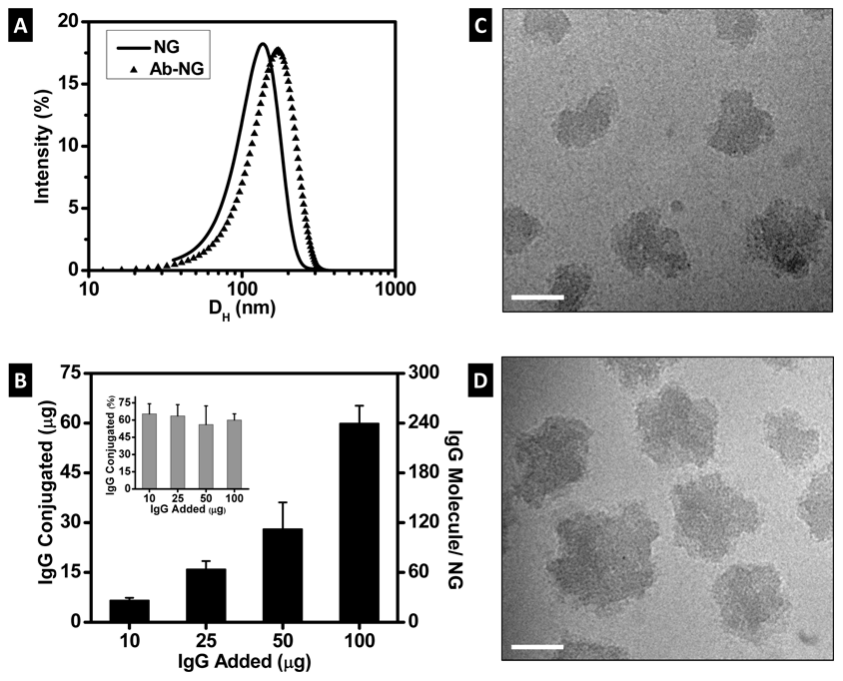
Characterization of Ab-NG. (a) Size distribution of nanogel before (NG) and after Ab coating (Ab-NG), as measured by DLS. The particle size (z-average) is 137 nm (pdi = 0.081) and 160 nm (pdi = 0.095) for NG and Ab-NG, respectively. (b) Summary of anti-ICAM coverage of NGs, [^125^I]IgG conjugation increases with [^125^I]IgG added to the NGs. Representative cryo-TEM images of NG before (c) and after (d) anti-ICAM coating (ICAM-NG). Scale bar: 50 nm.

A schematic describing the steps for conjugation of Ab to NG and loading of DEX is given in Figure 1. For Ab conjugation, the dextran present on the outer layer of the NG was oxidized with a reducing agent, NaIO_4_, to yield reactive formyl groups. These formyl groups were then reacted with Ab molecules via primary amine groups. The latter reaction was carried out at 4°C to minimize Ab degradation and/or inactivity. Independent of Ab conjugation, the NGs diameters displayed Gaussian distributions as measured by DLS (Figure 2a). The particle size (z-average) increased 17% upon Ab conjugation, from 137 nm (pdi = 0.081) to 160 nm (pdi = 0.095). By volume distribution, similar particle sizes were observed (139±47 for NG, 173±61 for Ab-NG) suggesting modal distribution of particles. Considering the hydrodynamic radius of IgG is 5.3 nm [39], the NG size increased as expected with Ab conjugation.

To target the pulmonary vasculature, NGs were coated with Ab directed to endothelial determinant ICAM-1(ICAM-NG) or control IgG (IgG-NG). The amount of Ab conjugated to the NG (Figure 2b) was quantified by gamma radiation using radiolabeled [^125^I]IgG. The Ab conjugated to the NG was optimized and reaction efficiency of 60% IgG conjugation was observed within the concentrations of IgG studied (10 to 100 µg). At the highest IgG concentration studied, NG were decorated with ∼250 Ab molecules per NG.

The morphology of the nanogels in the hydrated state, prior (NG) and subsequent to anti-ICAM conjugation (ICAM-NG), was visualized by cryo-TEM (Figure 2c–d). The nanogels were well-dispersed and irregularly shaped, consistent with lightly crosslinked (i.e., highly hydrated) structures. The average size of the hydrated nanogels observed by TEM was significantly smaller than the hydrodynamic diameter measured by DLS. These divergences are explained based on both techniques limitations. On one side, DLS assumes transparent solutions of diluted spherical colloids. However, the nanogels used in this study, scatter, fluoresce and are irregularly shaped. Moreover, the hydrodynamic diameter measured by DLS, corresponds to the diameter of the nanogels plus the thickness of its solvation layer and therefore, can easily overestimate diameters. By comparison, cryo-TEM is based on electron density contrast particularly near the periphery of the nanogel and therefore poor electron density contrast of the nanogel due to a highly swollen network can underestimate diameters [40].

### In vivo targeting of [^125^I]Ab-NG to pulmonary vasculature

**Figure 3:**
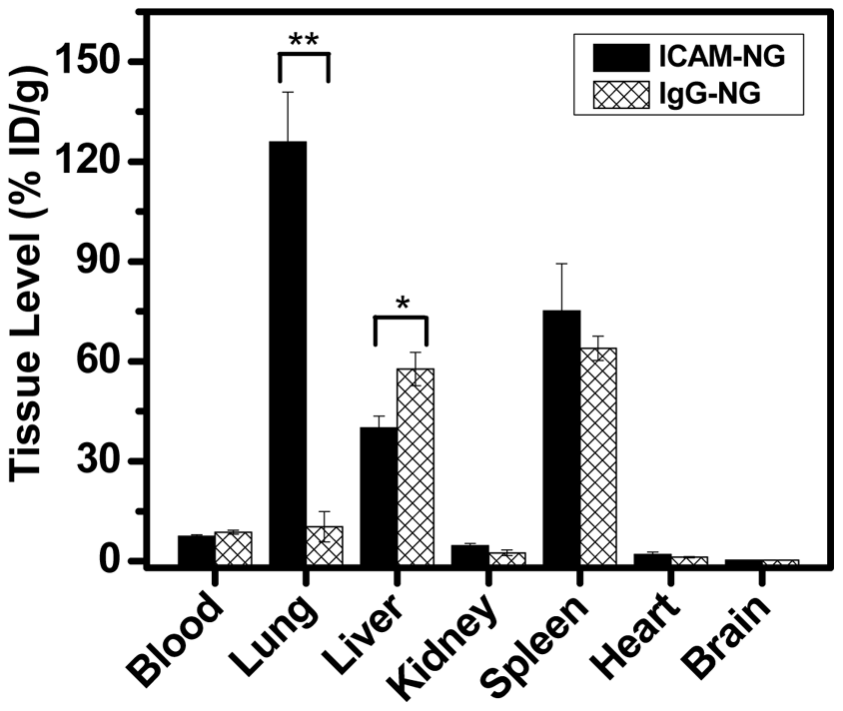
*In vivo* targeting of [^125^I]Ab-NG. Organ uptake of [^125^I]ICAM-1 targeted and [^125^I]IgG-NG were analyzed at 30 min post-IV administration. **p<0.005, *p<0.05.

*In vivo* biodistribution analyses confirmed that the anti-ICAM conjugated to NG (ICAM-NG) was still bioactive (Figure 3). Analyses of different tissues and blood after injection of ICAM-NG targeted to endothelial cells showed preferential location (∼12-fold increase in tissue level) of the NG in the pulmonary vasculature compared to non-specific control experiments (IgG-NG). At lower extent, ICAM-NG and IgG-NG were also taken up by organs in the mononuclear phagocyte system, such as the liver and the spleen. Interestingly, the hepatic uptake of ICAM-NG was markedly lower (30%) as compared to IgG-NG, indicating that specific targeting to endothelium, first of all, in the lungs, competes successfully with non-specific uptake in the mononuclear phagocyte system. A more accurate measure of NG targeting efficiency can be obtained by comparing the specific targeting (ICAM-NG) relative to non-specific targeting (IgG-NG) of the nanogels to the target (lungs) versus a clearance organ (liver). By this measurements, the targeting efficiency of ICAM-NG to the lungs was 18-fold that of the liver. Furthermore, blood and other organs studied showed minimum Ab-NG uptake (<10%).

### Loading of DEX and in vitro release

**Figure 4:**
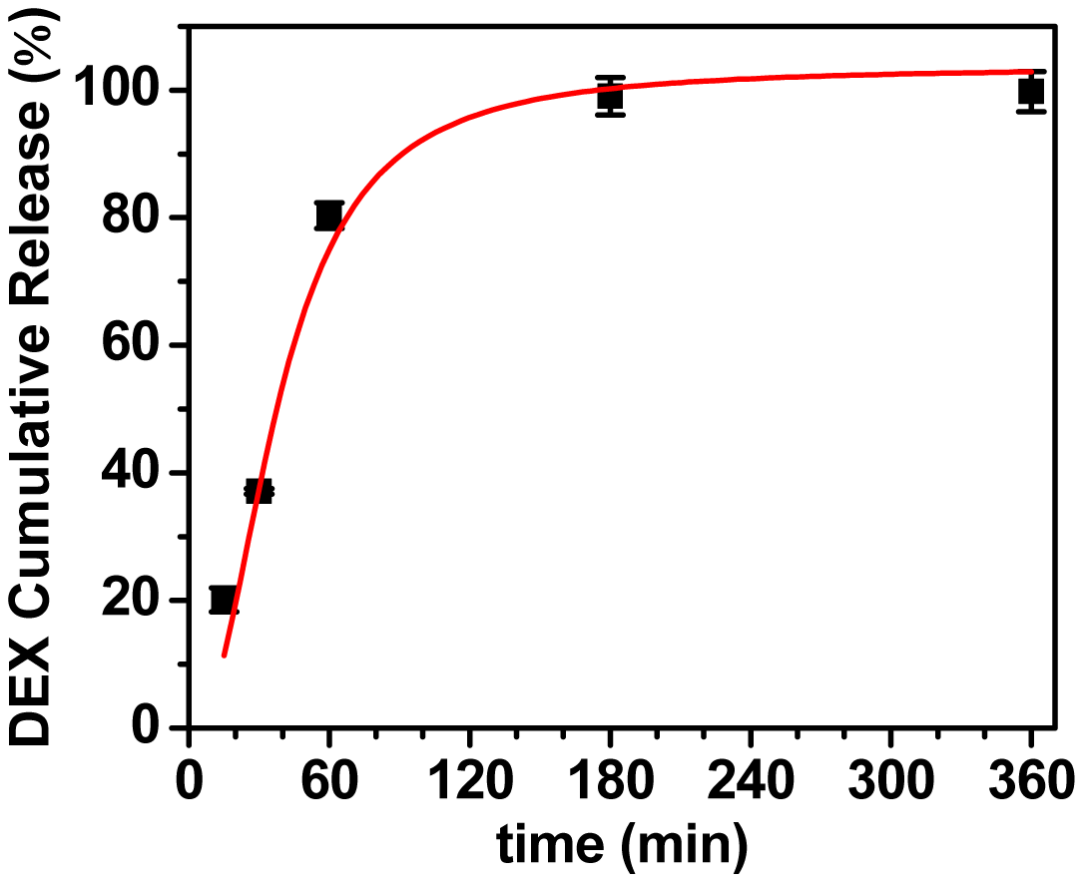
*In vitro* DEX release kinetics of IgG-NG in 1 wt% BSA/PBS. The red line is extended Langmuir model fit.

The Ab-NG were loaded 5% of their weight with DEX at 37°C. Significantly lower DEX payloads were measured when following attempts to load the drug at 4°C (data not shown). The *in vitro* release of DEX (at 37°C with agitation) is plotted in Figure 4 as accumulative release over time. An initial release burst was observed during the first hour as ∼80% of DEX was released. At longer time, DEX released slower, reaching complete release within 3 h. The experimental data followed an extended Langmuir model, represented by:
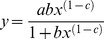
where a = 104, b = 3.1 E-4 and c = −1.2 (R^2^ = 0.95).

### In vivo administration of ICAM-NG-DEX in endotoxemia model mice

An endotoxemia model (LPS) was used to induce a systemic inflammatory response in mice. This challenge resulted in approximately 3-fold elevation of expression of an inflammatory marker, ICAM-1, in the inflamed pulmonary vasculature after 24 h (data not shown), in agreement with the literature [37].

**Figure 5:**
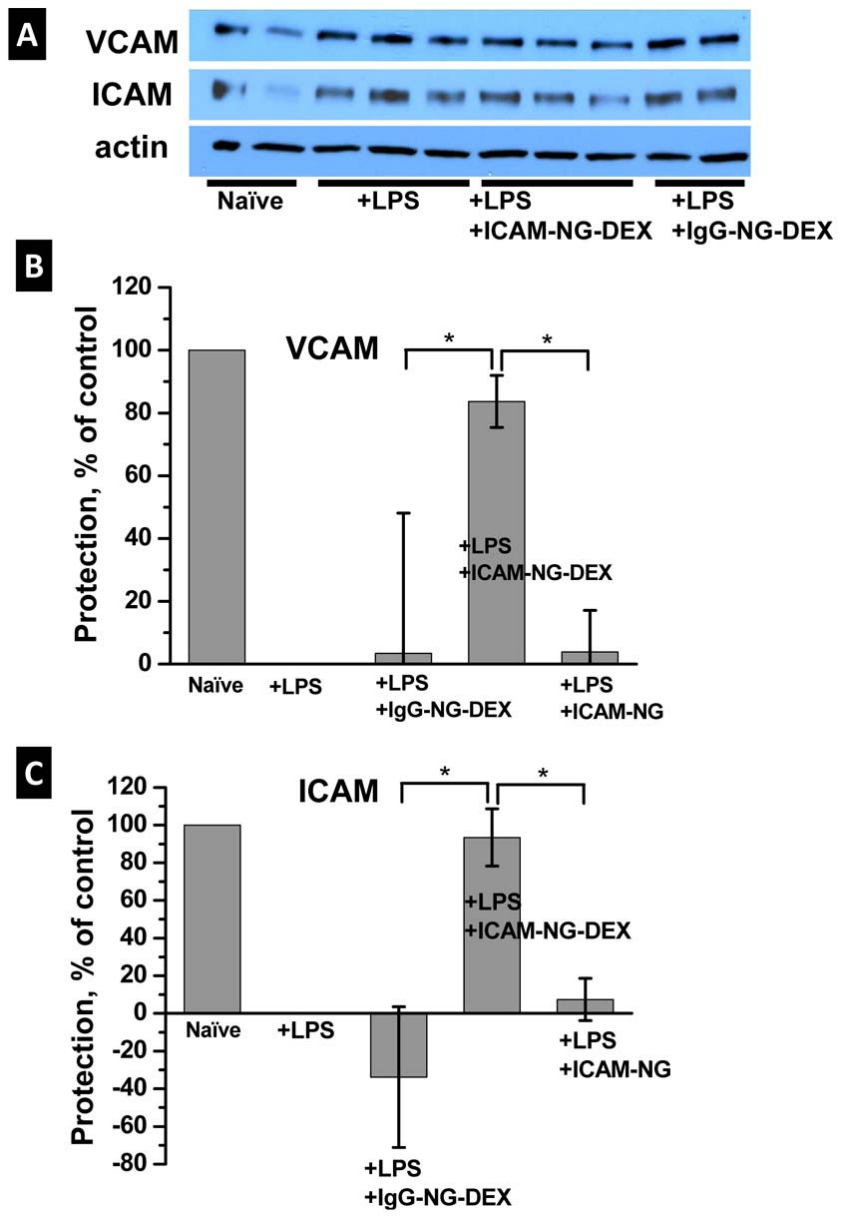
*In vivo* VCAM and ICAM protein expression in lung tissue for naïve and LPS-treated (+LPS) mice following injection for the different treatments studied. (a) Representative Western blot detection, (b) summary of % protection for VCAM expression, (c) summary of % protection for ICAM expression. *p<0.05.

The potential of ICAM-NG-DEX to reduce pulmonary vasculature inflammation in mice was measured as % protection based on the protein levels in the lungs of two inflammatory markers, ICAM-1 and VCAM-1, assessed 24 h after therapeutic injection (Figure 5). As controls, naïve mice (100% protection), bare endotoxemia model mice (+LPS, 0% protection) and endotoxemia model mice previously injected with non-specific NG loaded with DEX (+LPS, +IgG-NG-DEX) or specific NG with no drug (+LPS, ICAM-NG) were used. In the animals given the targeted, therapeutic-laden nanogels ICAM-NG-DEX, there was drastic reduction in pulmonary vasculature inflammation to levels found in naïve mice. In particular, injection of ICAM-NG-DEX provided 93% and 84% protection against inflammation expression of ICAM-1 and VCAM-1 in lung tissue, respectively. As expected, neither injection of NG loaded with DEX but lacking targeting, IgG-NG-DEX, nor injection of NG with targeting but lacking DEX, suppressed inflammation in mice.

## Discussion

In this study, we have developed for the first time NG targeted to the lungs by direct covalent immobilization of Ab against endothelium determinant, ICAM-1, using simple and direct chemistry (i.e., single step, no Ab modification required, Figure 1).

This novel methodology introduces advantages over ICAM-1 targeted nanocarriers prepared via electrostatic/hydrophobic interactions [37]. In our NGs, the dextran shell of the NG was activated to generate free aldehydes, for subsequent Ab immobilization via free amines. The covalent bond prevents Ab displacement by proteins and leaching off the surface, favors stability of the attached Ab (i.e., prevents denaturation) and allows for better control of the number/location of Ab binding sites [41]. Using this aldehyde-mediated direct chemistry we demonstrated conjugation of Ab onto NG by DLS, gamma radiation and cryo-TEM (Figure 2). In particular, we conjugated the NG with anti-ICAM to target the pulmonary endothelium or IgG to use as a control (i.e., non-specific targeting). Complete saturation of Ab (∼60%) at the highest concentration of IgG studied led to ∼240 Ab molecules per NG particle, which is consistent with our geometric estimates of ∼250 Ab molecules per NG particle indicated above. This surface coverage has shown 100% endothelium *in vivo* targeting with anti-ICAM molecules in mice when using polystyrene beads (100 nm) as a nanocarrier [42].

Importantly, the Ab in our ICAM-NG maintained their biological activity (Figure 3). This result suggests that possible drawbacks associated with Ab conjugation by direct chemistry are kept to a minimum (i.e., no site-specificity, uncontrolled Ab orientation, and need for a further step reduction of the imine bond to amine). Most importantly, we demonstrated that our ICAM-NG effectively targeted specific anatomical tissue/cells *in vivo* (i.e., pulmonary endothelium, Figure 3) in the resting state while showing low specificity for clearance tissues involved in the mononuclear phagocyte system (i.e., liver, spleen). Note that in the resting state, the levels of ICAM expressed by endothelial cells were about 3-fold lower than in the activated state. This result further highlights ICAM-NG extraordinary specificity. Relative to the liver, the amount of ICAM-NG that reached the lungs was 18-fold higher than IgG-NG.

As a proof of concept, the potential of ICAM-NG to upload and offload drugs via diffusion was tested using DEX. Ab-NG-DEX exhibited a fast onset of DEX action. A DEX upload of 5 wt% of the Ab-NG-DEX particle's own weight was released continuously and rapidly within 1 h (∼80% released) whereas the remainder of the drug was entirely released at a slower pace within 3 h (Figure 4), following an extended Langmuir model fit. This fast onset of DEX action is particularly important when used for prophylaxis in surgery to minimize postoperative inflammation, pain, nausea and vomiting [43]. A single preoperative dose of DEX (∼8 to 10 mg/kg in adults) has shown benefits in various surgeries [44], such as cardiac [45] and thyroid operations [46]. However, free drug has also been associated with toxicity to non-target organs. Alternatively, drugs can be delivered specifically using targeted nanocarriers, which would, ideally, maintain the therapeutic efficiency of the drug while reducing its toxicity to other organs [47]. We chose endothelium determinant ICAM-1 because is expressed in the rested and pathological activated state [48]. Therefore, our ICAM-NG-DEX can be administered prophylactically to prevent inflammation from occurring.

*In vivo* studies demonstrated the therapeutic efficiency of ICAM-NG-DEX in mice. These NG targeted to the lungs succeeded to deliver DEX, as indicated by the low levels of pro-inflammatory cytokines measured in the lungs of endotoxemic mice pretreated with ICAM-NG-DEX (Figure 5). The importance of targeting the drug-loaded NG is further confirmed by the lack of action to minimize lung inflammation in endotoxemic mice when IgG-NG-DEX is used instead of ICAM-NG-DEX or when DEX is not loaded in the NG (ICAM-NG).

These results highlight the potential of our NGs to selectively deliver drug intracorporeally by intravascular injection. By changing the Ab, this methodology can be easily extended to prepare NGs aimed at other targets (i.e., cells types, tissue, organs). As a proof of concept, we have demonstrated that the NG can effectively deliver a drug, in this case the anti-inflammatory steroid DEX, to the target cells while minimizing drug offloading into the other organs, and minimizing early drug loss via clearance mechanisms involving the RES. Future studies will focus on optimization of uptake/offloading of different drugs into these NG.

## Conclusions

A novel approach to prepare nanogels targeted to the lungs is demonstrated by conjugating them with anti-ICAM antibodies. These NG loaded with dexamethasone and administered prophylactically successfully minimize inflammatory response in acute lung injury in endotoxemia model mice.
